# Serum Symmetric Dimethylarginine as an Early Marker of Excretory Dysfunction in Canine Leishmaniosis* (L. infantum)* Induced Nephropathy

**DOI:** 10.1155/2018/7517359

**Published:** 2018-05-13

**Authors:** Esther Torrent, Marta Planellas, Laura Ordeix, Josep Pastor, Jaume Rodon, Laia Solano-Gallego

**Affiliations:** ^1^Fundació Hospital Clínic Veterinari, Universitat Autònoma de Barcelona, Barcelona, Spain; ^2^Departament de Medicina i Cirurgia Animals, Facultat de Veterinària, Universitat Autònoma de Barcelona, Barcelona, Spain; ^3^IDEXX Laboratorios, Barcelona, Spain

## Abstract

The aims of the study were to determine whether symmetric dimethylarginine (SDMA) was increased in dogs with leishmaniosis and to assess its relationship with creatinine concentration and urinary protein : creatinine ratio (UPC) to determine its utility as a marker of early excretory dysfunction. Fifty-three dogs with leishmaniosis classified according to the LeishVet clinical staging (stage I, *n* = 5, stage II, *n* = 30; stage III, *n* = 12; stage IV, *n* = 6) were selected and compared with 41 clinically healthy dogs. Thirty-nine dogs with leishmaniosis were also followed up for six months. SDMA concentrations on the day of diagnosis were significantly higher in dogs with leishmaniosis with respect to control dogs and in dogs from LeishVet stage IV when compared with the other stages. Increased UPC (>0.5), SDMA (>19 *μ*g/dL), and creatinine concentrations (≥1.4 mg/dL) were found in 47.1%, 15.1%, and 9.4% of dogs with leishmaniosis, respectively. SDMA concentration was increased in 24% of proteinuric dogs, in 7% of nonproteinuric dogs, and in four of five dogs with increased creatinine. SDMA concentration ≥ 25 *μ*g/dL was associated with clinical chronic kidney disease (CKD) after six months. Our results did not demonstrate advantages in using SDMA concentration as an early marker of CKD when compared to creatinine and UPC in canine leishmaniosis.

## 1. Introduction

Canine leishmaniosis (CanL) is a widely distributed protozoal disease that, in the Mediterranean basin, is caused by* Leishmania infantum* and transmitted by phlebotomine sand flies [1]. Infected dogs show a variable range of clinical manifestations depending on the type of immune response developed against the parasite [1–3]. Some dogs with predominant cell mediated immunity may remain subclinically infected [2], while those that develop a predominantly nonprotective parasite specific humoral response tend to progress to clinical illness [2, 4, 5].

Dogs with an exacerbated humoral response and hyperglobulinemia can have deposition of circulating immune complexes at the glomerular level, inducing inflammatory changes that lead to glomerular damage [4, 6–8]. Histopathology of renal lesions in CanL commonly reveals glomerulonephritis and tubulointerstitial nephritis [8, 9], although some authors consider that tubulointerstitial lesions appear secondarily to glomerular pathology due to inflammation and fibrosis of renal interstitium [10]. Initial glomerulonephritis can manifest as asymptomatic proteinuria [8, 11], but as the proteinuric nephropathy progresses, it can lead to excretory dysfunction with increased or decreased glomerular filtration rate (GFR) [9, 12] and to systemic hypertension. Increased GFR and hypertension can amplify the glomerular pathology resulting in progression of chronic kidney disease (CKD) [8]. Development of end stage CKD is a severe manifestation of disease progression and the principal cause of death in CanL [8, 13]. However, improvement of azotemia and proteinuria depending on treatment and severity of the disease has been described [9, 14, 15]. The two main parameters used to classify the degree of severity of renal disease in dogs with leishmaniosis according to the LeishVet clinical staging system are the urinary protein : creatinine ratio (UPC) as a marker of glomerular pathology and creatinine concentrations as a marker of excretory renal function [16].

SDMA is a methylated arginine produced by cellular protein catabolism [17] that has demonstrated a good correlation with creatinine and with GFR in dogs [18]. Several studies have shown that SDMA is elevated in dogs with CKD [18, 19] and with acute kidney injury [20]. Moreover, in CKD, SDMA seems to increase earlier that creatinine [18, 19] and is not influenced by dogs' muscle mass [21], or inflammatory states in humans [22, 23].

Recently, a new technique for the routine detection of canine SDMA, namely, the IDEXX SDMA® test, has been developed and validated [24], and interpretation of SDMA concentrations has been included in the International Renal Interest Society (IRIS) CKD staging guidelines [25].

Since renal disease might develop in dogs with leishmaniosis, feasible diagnostic tools, which might improve the detection of decreased renal function, would be helpful for the practicing veterinarian. Therefore, we proposed a retrospective descriptive study with the following specific objectives: (1) to determine serum SDMA concentrations in dogs in different clinical stages of leishmaniosis [16] at the time of diagnosis, (2) to evaluate the correlation of SDMA concentrations with serum creatinine concentrations and UPC, and (3) to describe the outcome of dogs with leishmaniosis during six months after diagnosis.

## 2. Materials and Methods

### 2.1. Dogs and Serum Samples

Residual frozen serum samples kept at −80°C from 53 dogs with leishmaniosis and 41 healthy age-matched dogs were used for this study.

Dogs had been diagnosed with leishmaniosis at five veterinary centers from the northeast of Spain between January 2014 and December 2016. On the day of diagnosis, all dogs had undergone physical examination and had laboratory tests performed. The laboratory tests consisted of complete blood cell count, biochemistry panel (with measurement of urea, creatinine, cholesterol, alanine transaminase activity, and total proteins concentrations), serum protein electrophoresis, an endpoint quantitative serology for the detection of* L. infantum* specific antibodies by means of an ELISA in-house [26], and a complete urinalysis with UPC determination. Dogs with urinary infection and those without UPC performed on the day of diagnosis were excluded from the study.

Diagnosis of leishmaniosis was based on compatible clinical and clinicopathological findings along with high serological antibodies levels or, in some cases, visualization of the parasite in cytological samples from lymph node, skin, or bone marrow. Occasionally, histopathological and immunohistochemistry [27] identification of* Leishmania *spp. in cutaneous lesions was also performed.

According to the results of their physical examination and laboratory tests, dogs were classified into the following clinical stages: I (*n* = 5), II (*n* = 30), III (*n* = 12), and IV (*n* = 6), as described by the LeishVet clinical staging system [16] (Supplementary Table 1).

Healthy control dogs were owned by staff from several of the veterinary centers involved in the study, or were brought for routine health check or for sterilization. The inclusion criteria for these dogs were (1) an unremarkable physical examination, (2) a seronegative result by an in-house ELISA for the detection of antibodies against* L. infantum *antigen [26], and (3) no abnormalities in routine biochemistry or hematological profile.

### 2.2. Follow-Up of Dogs with Leishmaniosis during Six Months

**Figure 1:**
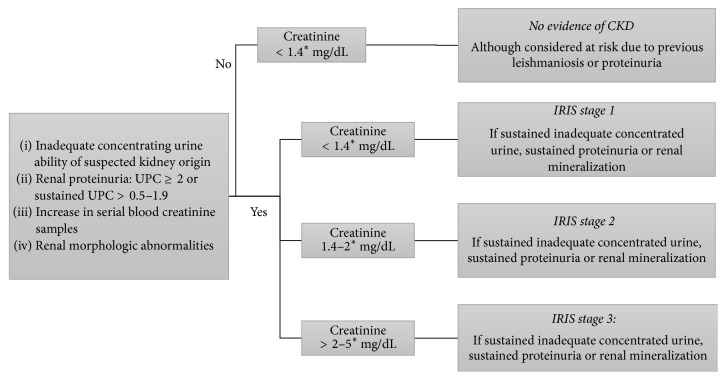
Algorithm for classifying dogs with leishmaniosis after six months of diagnosis according to the presence of CKD based on IRIS staging system [25]. IRIS: International Renal Interest Society; CKD: chronic kidney disease; UPC: urinary protein/creatinine ratio. ^*∗*^Blood creatinine concentrations applied to average sized dogs. Values ≥ 1.4 mg/dL were present more than once during the follow-up.

Dogs with leishmaniosis had their medical records reviewed to gather information about their clinical evolution during the first six months following the diagnosis. Data about their history, clinical signs, clinicopathological and serological tests, urinalysis, UPC determination, and ultrasound abnormalities were recorded. According to this information, treated dogs were classified into two groups (Group A and Group B) based on their clinical outcome and renal disease status six months after diagnosis. Nontreated patients, dogs that developed other diseases during the six months or had insufficient tests performed to assess kidney function, were excluded from the classification. Dogs with well-controlled leishmaniosis that had no clinical signs or clinicopathological abnormalities suggesting CKD (including creatinine concentration < 1.4 mg/dL, UPC ≤ 0.5, adequate concentrating ability measured by refractometry, and absence of renal abnormalities on physical examination or ultrasound imaging) were assigned to Group A. Group B included dogs with CKD as defined by IRIS staging guidelines [25] and dogs that died during the follow-up period due to renal disease. An algorithm showing how IRIS staging guidelines were applied for the classification of dogs is shown in Figure 1.

### 2.3. SDMA, Creatinine, and UPC Analysis

UPC was measured on the day of diagnosis on urine samples with inactive sediment. Urine samples collected by free catch or cystocentesis were centrifuged and urinary protein was measured using pyrogallol red combined with molybdate (Beckman Coulter AU400) and urinary creatinine using the Jaffé method (Beckman Coulter AU400). Dogs were considered proteinuric when UPC was >0.5.

Banked serum samples were thawed and sent under refrigeration to IDEXX Laboratories (Spain) for measurement of SDMA and creatinine concentrations. SDMA concentration was measured using the IDEXX SDMA Test (Beckman Coulter AU640) which is based on a previously validated [24] immunoassay using a glucose-6-phosphate dehydrogenase conjugate and an anti-SDMA monoclonal antibody [28]. Creatinine concentration was measured using the Jaffé method [29] (Beckman Coulter AU640). Creatinine concentrations ≥ 1.4 mg/dL were considered increased according to the IRIS and LeishVet classifications. Based on IDEXX Laboratories algorithm for interpreting one-single-point SDMA measurements [30], a medical decision cut-off point of >19 *μ*g/dL was used to define increased concentrations of SDMA.

### 2.4. Statistical Analysis

Statistical analysis was performed using a commercial software program (IBM SPSS® Statistics version 22). Parameters were investigated for normality using the Kolmogorov-Smirnov test and data were analyzed using nonparametric statistical methods. Correlation between creatinine, SDMA, and UPC was measured using Spearman's correlation coefficient. Mann–Whitney *U* test was used to compare SDMA and creatinine concentrations, UPC, and age between dogs with leishmaniosis and control dogs and between Group A and Group B dogs, as well as SDMA concentrations between proteinuric and nonproteinuric dogs with leishmaniosis. SDMA concentration in dogs from different LeishVet stages was compared using the Kruskal-Wallis test followed by a post hoc comparison with a Bonferroni correction. Odds ratios and 95% confidence intervals for dogs belonging to Groups A and B were calculated according to the following cut-off values of several parameters measured on the day of diagnosis: (1) SDMA concentration > 19 *μ*g/dL and ≥25 *μ*g/dL, (2) creatinine concentration ≥ 1.4 mg/dL, and (3) UPC > 0.5 and >5 compared with lower values. Fisher's exact test was used to calculate *p* values for odds ratios. Significant differences were defined as having *p* values < 0.05.

## 3. Results

### 3.1. Clinical Data at Diagnosis

Dogs with leishmaniosis included 25 males (three neutered) and 28 females (13 spayed). Thirty-six of the 53 dogs were purebred. The main breeds were distributed as follows: Boxer (*n* = 4), Labrador Retriever (*n* = 2), and Greyhound (*n* = 2). In addition, two dogs were from each of the following breeds: German Shepherd, German Shorthaired Pointer, Épagneul Breton, Doberman, Golden Retriever, and French Bulldog. Sixteen dogs from other different breeds were also included.

The healthy control group was composed of 20 males (two neutered) and 21 females (four spayed). There were three dogs of unknown breed, eleven mongrel dogs, four Greyhounds, three Golden Retrievers, three Ibizan Hounds, two Ariegeois, two Border Collies, two Teckels, two Pugs, two English Setters, and seven other dogs, each from a different breed.

The median age of dogs with leishmaniosis was 48 months (range from 5 to 156 months), while the median age of control dogs was also 48 months (from 6 to 132 months). No significant differences in age were observed between both groups (Mann–Whitney *U* test; *p* = 0.56).

**Figure 2:**
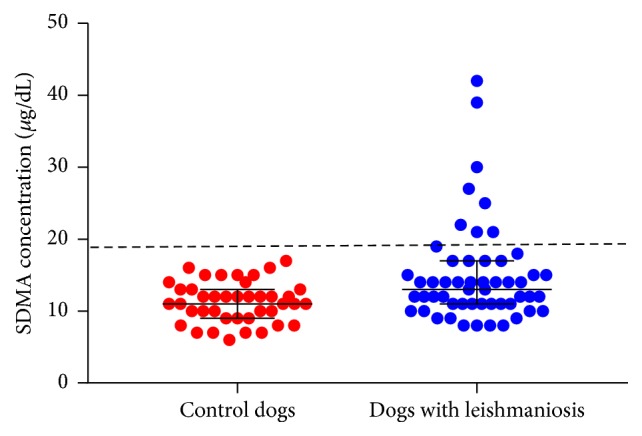
Distribution of SDMA concentrations in 53 dogs with leishmaniosis on the day of diagnosis (blue dots) and 41 control dogs (red dots). The large black bar indicates the median of each group and the whiskers indicate the interquartile range. The dashed line indicates SDMA concentration of 19 *μ*g/dL.

SDMA concentration of dogs with leishmaniosis (median = 13 *μ*g/dL, range = 8–42) was significantly higher than that of control dogs (median = 11 *μ*g/dL, range 6–17) (Mann–Whitney *U* test, *p* = 0.005).  Figure 2 shows the distribution of SDMA concentration in dogs with leishmaniosis and control dogs. SDMA concentration was >19 *μ*g/dL in 8/53 (15.1%) dogs with leishmaniosis while all control dogs were below this cut-off value. SDMA concentrations were from 15 to 17 *μ*g/dL in seven control dogs. In addition, SDMA concentrations were from 15 to 19 *μ*g/dL in nine dogs with leishmaniosis.

Five dogs with leishmaniosis (9.4%) had creatinine levels ≥ 1.4 mg/dL (range 1.5–3.6). All of them were proteinuric, and four of them had increased SDMA concentrations. The dog with normal SDMA concentration (18 *μ*g/dL) was a 36-month-old Greyhound with creatinine concentration of 1.8 mg/dL.

Dogs with leishmaniosis had median UPC of 0.4 (range 0.08–13.86), with 25/53 (47.1%) of them being proteinuric (UPC > 0.5). SDMA concentrations were >19 *μ*g/dL (from 21 to 42 *μ*g/dL) in 6/25 (24%) proteinuric dogs and in two of 28 (7.1%) nonproteinuric dogs (with SDMA concentrations of 22 *μ*g/dL and 30 *μ*g/dL). No significant differences (Mann–Whitney *U* test *p* = 0.086) were found between SDMA concentrations of nonproteinuric dogs (median 12 *μ*g/dL, range 8–30) with respect to proteinuric dogs (median 14 *μ*g/dL, range 8–42).

**Figure 3:**
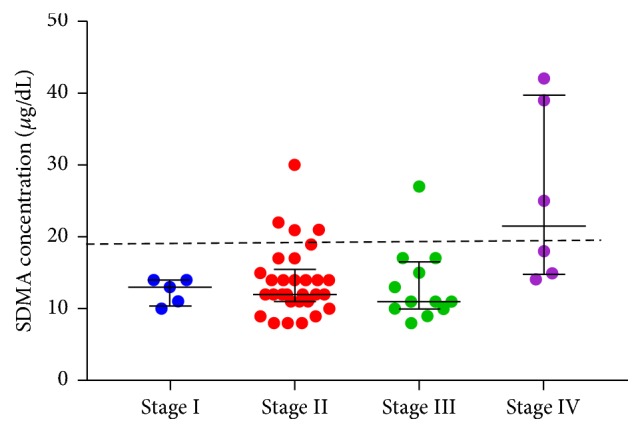
Distribution of SDMA concentrations in 53 dogs with leishmaniosis on the day of diagnosis classified according to LeishVet guidelines [16]. Stage I included 5 dogs, stage II 30 dogs, stage III 12 dogs, and stage IV 6 dogs. SDMA concentration was higher in dogs from stage IV with respect to stage I (*p* = 0.02), stage II (*p* = 0.006), and stage III (*p* = 0.004) dogs. The large black bar indicates the median of each group and the whiskers indicate the interquartile range. The dashed line indicates SDMA concentration of 19 *μ*g/dL.

Four of the eight dogs with increased SDMA concentration were classified in LeishVet stage II; two of them were not proteinuric and the other two had UPC between 0.5 and 1. One of the nonproteinuric dogs had SDMA concentration of 30 *μ*g/dL, while the other three had SDMA concentrations between 21 and 22 *μ*g/dL. The other four dogs had SDMA ≥ 25 *μ*g/dL. All of them were proteinuric and azotaemic and were classified into stage III (*n* = 1) and stage IV (*n* = 3).  Figure 3 shows SDMA distribution in dogs from the different LeishVet stages. No dogs in stage I had increased SDMA concentrations. SDMA concentrations varied depending on the LeishVet stage (Kruskal-Wallis *H* test; *X*^2^(3) = 10.28, *p* = 0.016). Post hoc analysis showed significant differences between SDMA concentrations of stage IV when compared with stage I (*p* = 0.02), stage II (*p* = 0.006), and stage III (*p* = 0.004) dogs. There were no differences in SDMA concentrations between the other stages.

SDMA concentration demonstrated a significant weak positive correlation with creatinine concentration (Spearman's rho = 0.386, *p* < 0.0001) and no significant correlation with UPC (rho = 0.243, *p* = 0.080). UPC did not show significant correlation with serum creatinine (rho = 0.056, *p* = 0.693).

### 3.2. Six-Month Follow-Up

One nonazotaemic and nonproteinuric dog with SDMA concentration of 30 *μ*g/dL died suddenly before starting treatment. Two dogs did not receive treatment, and two other dogs were lost to follow-up before the first month. The remaining dogs underwent treatment with a daily subcutaneous injection of meglumine antimoniate (80–100 mg/kg) for a month and allopurinol administered orally (10 mg/kg/12 hours) and were followed up for six months.

**Figure 4:**
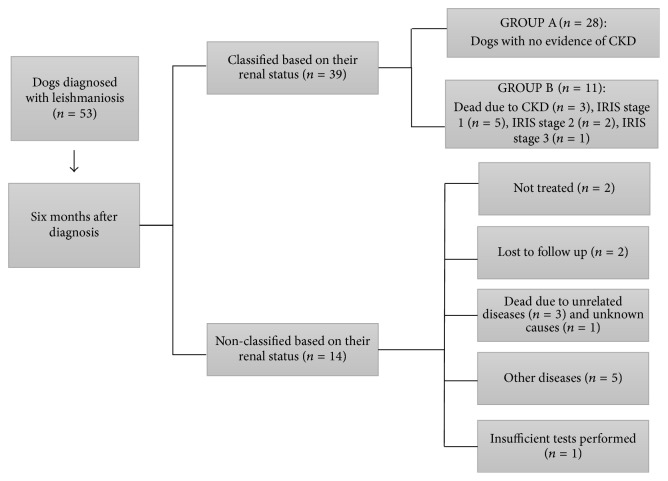
Flowchart depicting the clinical and renal outcome of 53 dogs with clinical leishmaniosis six months after diagnosis and their classification into two groups according to the presence of CKD. Group A: dogs with no evidence of CKD six months after diagnosis. Group B: dogs with CKD disease after six months of diagnosis or dogs that died due to renal disease during this period. Classification of IRIS stage was done following the IRIS guidelines for staging CKD [25] as detailed in Figure 1. IRIS: International Renal Interest Society; CKD: chronic kidney disease. *n*: number of dogs.

Two of the four dogs with initial SDMA concentration ≥25 *μ*g/dL that received treatment died before the first month due to CKD. The other two dogs were alive after six months but presented CKD IRIS stage 2 and IRIS stage 3. The other three dogs with increased SDMA (range 21-22 *μ*g/dL) did not show signs of CKD, and their SDMA normalized after six months of follow-up. All these three dogs had been initially classified as LeishVet stage II due to having UPC < 1.  Figure 4 summarizes the clinical and renal outcomes of the 53 dogs with leishmaniosis six months after diagnosis as well as their classification in Group A and Group B.

Age, UPC, and creatinine and SDMA concentrations at the time of diagnosis were compared between dogs from Group A and Group B, finding significant differences in all the parameters except for age (Table 1).

Table 2 shows the odds ratio for being classified in Group A* versus* Group B after six months of diagnosis based on different cut-off values of SDMA, creatinine, and UPC at diagnosis.

## 4. Discussion

Studies regarding the use of SDMA as a renal biomarker for kidney disease [18–20, 31–33] are sparse, and only one of them included nonazotaemic dogs with leishmaniosis [33]. To the best of the authors' knowledge, this study assessed for the first time SDMA concentrations in dogs with the four degrees of disease severity established by the LeishVet clinical staging system [16].

Glomerulonephritis is the main pathological event in leishmanial nephropathy [8], but severe glomerular pathology can lead to a variable reduction of GFR [9, 12]. In CanL, Plevraki et al. [9] found reduction of GFR in two of ten asymptomatic nonazotaemic proteinuric dogs, and Cortadellas et al. [12] in 11 of 26 dogs, most of them azotaemic and highly proteinuric. We also detected more leishmaniotic dogs with proteinuria (47.1%) than with increased SDMA (15.1%) and creatinine (9.4%) concentrations, which are markers of excretory dysfunction. The percentage of dogs with increased SDMA in our study was lower in comparison with these previous studies [9, 12], probably because of the inclusion of a higher number of nonproteinuric dogs from LeishVet stages I and II, which probably had no nephropathy or a very low degree of it. We found weak correlation between serum creatinine and SDMA concentration, in agreement with previous results in healthy dogs [21]. Creatinine and SDMA are endogenous biomarkers of renal excretory function; however, SDMA has been suggested to increase earlier than creatinine in CKD [18, 19]. In our study, we found a Greyhound with a mild increase in creatinine without elevated SDMA concentration. This result can be attributed to the idiosyncrasy of this breed, which presents higher creatinine concentrations [34] and probably SDMA levels when compared with dogs from other breeds [35].

We did not find correlation between UPC and creatinine or SDMA concentrations. The higher percentage of proteinuric dogs in comparison with dogs with increased SDMA or creatinine concentrations was expected as proteinuria has been described as the first clinicopathological finding indicating leishmanial nephropathy [8, 11], and not all dogs with glomerulonephritis have damage severe enough to affect excretory renal function. Besides, some proteinuric dogs can develop glomerular hyperfiltration [12] which could maintain normal SDMA concentration despite a higher degree of renal pathology. Occasionally, the presence of proteinuria in the absence of evident morphologic renal lesions has also been observed in CanL [36], and it is hypothesized that overload proteinuria due to serum excess of immunoglobulin free light chain proteins could be present in these dogs. However, in the present study, renal histopathology to confirm the absence of kidney lesions or urine immunoelectrophoresis to determine the origin of urinary proteins was not performed.

On the other hand, our study found two nonproteinuric dogs with elevated SDMA concentration and creatinine within reference limits. One of these dogs died unexpectedly while the other one showed no clinical evidence of renal disease after six months. Cortadellas et al. [12] found that one of eight dogs with CanL and UPC levels between 0.2 and 0.5 had decreased GFR. Some dogs with leishmaniosis can have an impairment of renal perfusion secondary to hypovolemia or severe dehydration which would lead to an increase in SDMA concentration not associated with proteinuria or renal azotemia. Vasculitis of renal arterioles [37, 38] and myocarditis [38–41] have also been described in dogs with leishmaniosis and should be taken into account as less probable causes of decreased GFR. We suggest that dogs with leishmaniosis and moderate to high increases in SDMA concentrations without proteinuria should be carefully evaluated for other diseases or pathogenic mechanisms which could lead to prerenal impairment of renal perfusion.

We only found significant differences in SDMA concentrations in dogs from LeishVet stage IV with respect to the other three stages. We did not find a higher percentage of dogs with increased SDMA in stage III when compared with stage II as would have been expected due to the progressive increase in disease severity. Therefore, these results do not support the use of SDMA concentration for a better classification of severity in leishmaniosis, as dogs with stage IV have azotaemia and/or UPC > 5.

The prognosis for dogs with leishmaniosis is highly dependent on kidney function [16]. Improvement in kidney parameters such as UPC and creatinine levels after treatment has been described in dogs with leishmaniosis [9, 15]. In fact, some of the dogs in our study with initial proteinuria and increased SDMA did not show clinicopathological signs of renal disease after six months. In the present study, high odds ratios for presenting CKD or being dead after six months were found for creatinine ≥ 1.4 mg/dL, SDMA ≥ 25 *μ*g/dL, and UPC > 5. These cut-off values are probably associated with more severe kidney disease and less reversible kidney damage. In contrast, SDMA concentrations higher than 19 *μ*g/dL were not associated with an increased probability for presenting CKD, probably reflecting a low degree of renal damage. All dogs with initial SDMA concentration ≥ 25 *μ*g/dL died or presented CKD IRIS stage 2 or 3 after six months of follow-up. In agreement with the present results, SDMA concentrations higher than 25 *μ*g/dL have been recommended by the IRIS staging guidelines to classify dogs with low body condition scores as IRIS stage 3 despite having creatinine concentrations in the stage 2 range [25]. However, further studies with larger numbers of dogs would be encouraged to determine if SDMA concentration could be used as a prognosis marker for dogs with leishmaniosis and other renal diseases.

Previous studies evaluating SDMA used the upper reference limit (URL) of 14 *μ*g/dL provided by IDEXX Laboratories [18, 32, 33]. In our study, seven of 41 control dogs considered healthy had SDMA between 15 and 17 *μ*g/dL. For dogs with no suspicion of renal disease, repeating single SDMA measurement between 15 and 19 *μ*g/dL is recommended [30], but this was not possible due to the retrospective nature of our study. Some authors also have suggested that young dogs and Greyhounds might have a URL higher than 14 *μ*g/dL [18, 36], and in a recent study [42], the mean SDMA concentration of 20 apparently healthy dogs (12.7 *μ*g/dL) was situated in the top quarter of the population-based reference interval established by IDEXX Laboratories. Therefore, in order to reduce the number of dogs with doubtful SDMA classification (range 15–19 *μ*g/dL), we decided to use SDMA >19 *μ*g/dL as a clinical decision cut-off value for both control dogs and dogs with leishmaniosis to increase the specificity of the test in spite of a decrease in sensitivity.

## 5. Conclusions

Based on the present study results, the use of SDMA in dogs with leishmanial nephropathy has not shown advantages in comparison with creatinine concentrations and UPC for predicting renal failure or for a better differentiation in LeishVet stages. However, SDMA might be useful for detecting excretory dysfunction in dogs with leishmaniosis by other pathogenic mechanisms. Further studies investigating SDMA concentrations in leishmaniosis and other diseases with kidney involvement, as well as the association between different cut-off values for medical decision limit of SDMA and clinical outcome of dogs, are needed.

## Figures and Tables

**Table 1:** Comparison of age, creatinine, and SDMA concentrations and UPC on the day of diagnosis of leishmaniosis for Group A *versus* Group B dogs.

	Group A (median and range)	Group B (median and range)	*p*
	*N* = 28	*N* = 11	
Age (months)	42 (5–120)	62 (23–156)	0.14
Creatinine (mg/dL)	0.9 (0.7–1.3)	1.3 (0.8–3.6)	0.01
SDMA (*µ*g/dL)	12 (8–22)	17 (9–42)	0.018
UPC	0.28 (0.08–3.19)	4.15 (0.17–13.9)	<0.0001

Group A: dogs with no evidence of renal disease based on the IRIS staging for CKD [25] after six months of diagnosis of leishmaniosis. Group B: dogs with CKD classified as IRIS stage 1 to stage 3 and dogs euthanized or dead due to renal disease after six months of diagnosis of leishmaniosis. *p*: significance level (Mann–Whitney *U* test). *N*: number of dogs; UPC: urinary protein/creatinine ratio.

**Table 2:** Odds ratio for being classified in Group A or Group B after six months of follow-up depending on several cut-off values of kidney parameters on the day of diagnosis in 39 dogs with leishmaniosis.

Values on the day of diagnosis	Odds ratio^**∗**^ for being classified in Group A	Odds ratio^**∗**^ for being classified in Group B	*p*
SDMA > 19 *µ*g/dL	0.21 (0.04–1.16)	4.76 (0.85–26.47)	0.074
SDMA ≥ 25 *µ*g/dL	0.03 (0.001–0.605)	34.2 (1.65–708.19)	0.022^*∗∗*^
UPC > 0.5	0.047 (0.005–0.429)	21.11 (2.33–191.17)	0.0067^*∗∗*^
UPC > 5	0.03 (0.001–0.605)	34.2 (1.65–708.19)	0.022^*∗∗*^
Creatinine ≥ 1.4 mg/dL	0.021 (0.001–0.424)	48.23 (2.35–986.19)	0.00118^*∗∗*^

^**∗**^Expressed as odds ratio and its 95% confidence interval. ^*∗∗*^Significant value. Group A: dogs with no evidence of renal disease based on the IRIS staging for CKD [25] after six months of diagnosis of leishmaniosis. Group B: dogs with CKD classified as IRIS stage 1 to stage 3 and dogs euthanized or dead due to renal disease after six months of diagnosis of leishmaniosis. *p*: significance level (Mann–Whitney *U* test). UPC: urinary protein/creatinine ratio.

## Data Availability

The datasets used during the current study are available from the corresponding author on reasonable request.

## Supplementary Materials

Supplementary Materials A table titled “Supplementary Table 1” is included depicting the criteria based on LeishVet staging guidelines that have been used for the clinical staging of dogs with leishmaniosis in this study.
