# Supplementary material for: Serum Symmetric Dimethylarginine as an Early Marker of Excretory Dysfunction in Canine Leishmaniosis (L. infantum) Induced Nephropathy
Source: Vet Med Int. 2018 May 13;2018:7517359. doi: 10.1155/2018/7517359 (PMC5971233; doi:10.1155/2018/7517359)
Supplement: Supplementary Materials — A table titled “Supplementary Table 1” is included depicting the criteria based on LeishVet staging guidelines that have been used for the clinical staging of dogs with leishmaniosis in this study. [file 7517359.f1.zip › Supplementary material_VMI_2238292.docx]

**Supplementary Table 1.**

Clinical staging of canine leishmaniosis and relationship with prognosis according to LeishVet staging guidelines [16]

| Clinical stage | Serology | Clinical signs | Laboratory findings | Prognosis |
| --- | --- | --- | --- | --- |
| Stage I: mild disease | Negative to low positive antibody levels | Mild clinical signs (peripheral lymphadenopathy) or papular dermatitis | Usually no clinicopathological abnormalities observed. Normal renal profile: creatinine < 1.4 mg/dL, UPC < 0.5 | Good |
| Stage II: moderate disease | Low to high positive antibody levels | Apart from signs listed in stage I may present cutaneous lesions, anorexia, weight loss, fever or epistaxis. | Mild non-regenerative anemia, hypergammaglobulinemia, hypoalbuminemia.  Substage a: Normal renal profile: creatinine < 1.4 mg/dL, UPC < 0.5  Substage b: Creatinine < 1.4 mg/dL, UPC: 0.5-1 | Good to guarded |
| Stage III: severe disease | Low to high positive antibody levels | Apart from signs listed in stages I and II may present signs derived from immune-complex lesions. | Clinicopathological abnormalities listed in stage II. CKD IRIS stage I with UPC > 1 or stage II (creatinine: 1.4-2 mg/dL) | Poor to guarded |
| Stage IV: very severe disease | Low to high positive antibody levels | Clinical signs listed in stage III. Pulmonary thromboembolism, or nephrotic syndrome and end stage renal disease | Clinicopathological abnormalities listed in stage II. CKD IRIS stage III (creatinine 2-5 mg/dL) and stage IV (creatinine > 5mg/dL)  Nephrotic syndrome: UPC > 5 | Guarded |

CKD: chronic kidney disease. IRIS: international renal interest society. UPC: urinary protein ratio
