# Supplementary material for: Serum Symmetric Dimethylarginine as an Early Marker of Excretory Dysfunction in Canine Leishmaniosis (L. infantum) Induced Nephropathy
Source: Vet Med Int. 2018 May 13;2018:7517359. doi: 10.1155/2018/7517359 (PMC5971233; doi:10.1155/2018/7517359)
Supplement: Supplementary Materials — A table titled “Supplementary Table 1” is included depicting the criteria based on LeishVet staging guidelines that have been used for the clinical staging of dogs with leishmaniosis in this study. [file 7517359.f1.zip › supplementary material_VMI_2238293.pdf]

Supplementary Table 1.

Clinical staging of canine leishmaniosis and relationship with prognosis according to LeishVet staging guidelines [16]

Clinical stage	Serology	Clinical signs	Laboratory findings	Prognosis
**Stage I: mild disease**	Negative to low positive antibody levels	Mild clinical signs (peripheral lymphadenopathy) or papular dermatitis	Usually no clinicopathological abnormalities observed. Normal renal profile: creatinine < 1.4 mg/dL, UPC < 0.5	Good
**Stage II: moderate disease**	Low to high positive antibody levels	Apart from signs listed in stage I may present cutaneous lesions, anorexia, weight loss, fever or epistaxis.	Mild non-regenerative anemia, hypergammaglobulinemia, hypoalbuminemia. Substage a: Normal renal profile: creatinine < 1.4 mg/dL, UPC < 0.5 Substage b: Creatinine < 1.4 mg/dL, UPC: 0.5-1	Good to guarded
**Stage III: severe disease**	Low to high positive antibody levels	Apart from signs listed in stages I and II may present signs derived from immune-complex lesions.	Clinicopathological abnormalities listed in stage II. CKD IRIS stage I with UPC > 1 or stage II (creatinine: 1.4-2 mg/dL)	Poor to guarded
**Stage IV: very severe disease**	Low to high positive antibody levels	Clinical signs listed in stage III. Pulmonary thromboembolism, or nephrotic syndrome and end stage renal disease	Clinicopathological abnormalities listed in stage II. CKD IRIS stage III (creatinine 2-5 mg/dL) and stage IV (creatinine > 5mg/dL) Nephrotic syndrome: UPC > 5	Guarded

CKD: chronic kidney disease. IRIS: international renal interest society. UPC: urinary protein ratio
